# RhoA-Dependent HGF and c-Met Mediate Gas6-Induced Inhibition of Epithelial–Mesenchymal Transition, Migration, and Invasion of Lung Alveolar Epithelial Cells

**DOI:** 10.3390/biom9100565

**Published:** 2019-10-04

**Authors:** Jihye Jung, Kyungwon Yang, Hee-Ja Kim, Ye-Ji Lee, Minsuk Kim, Youn-Hee Choi, Jihee Lee Kang

**Affiliations:** 1 Department of Physiology, College of Medicine, Ewha Womans University, Seoul 07804, Korea; wowow0523@naver.com (J.J.); kwyang@ewhain.net (K.Y.); hjkim916@ewha.ac.kr (H.-J.K.); shyzizibe@naver.com (Y.-J.L.); yc@ewha.ac.kr (Y.-H.C.); 2 Tissue Injury Defense Research Center, College of Medicine, Ewha Womans University, Seoul 07804, Korea; ms@ewha.ac.kr; 3 Department of Pharmacology, College of Medicine, Ewha Womans University, Seoul 07804, Korea

**Keywords:** growth arrest-specific protein 6, hepatocyte growth factor, c-Met, epithelial-mesenchymal transition, lung epithelial cells

## Abstract

Previously, we demonstrated that growth arrest-specific protein 6 (Gas6)/Axl or Mer signaling inhibited the transforming growth factor (TGF)-β1-induced epithelial–mesenchymal transition (EMT) in lung epithelial cells. Hepatocyte growth factor (HGF) has also been shown to inhibit TGF-β1-induced changes in EMT markers. Here, we examined whether Gas6 signaling can induce the production of HGF and c-Met in lung alveolar epithelial cells to mediate the inhibition of EMT and to inhibit the migration and invasion of epithelial cells. The inhibition of the RhoA/Rho kinase pathway, using either a *RhoA*-targeted small interfering RNA (siRNA) or the Rho kinase pharmacologic inhibitor Y27362, prevented the inhibition of TGF-β1-induced EMT in LA-4 cells and primary alveolar type II (AT II) epithelial cells. The c-Met antagonist PHA-665752 also blocked the anti-EMT effects associated with Gas6. Moreover, treatment with Y27362 or PHA-665752 prevented the Gas6-mediated inhibition of TGF-β1-induced migration and invasion. Our data provided evidence that the RhoA-dependent production of HGF and c-Met mediated the Gas6-induced inhibition of EMT, migration and invasion in lung alveolar epithelial cells. Thus, Gas6/Axl and Mer/RhoA signaling may be necessary for the maintenance of homeostasis in the alveolar epithelium, via HGF and c-Met.

## 1. Introduction

Pulmonary fibrosis is a potentially fatal disease that is characterized by continuous alveolar epithelial injury and dysregulated repair, leading to myofibroblast accumulation and the excessive deposition of extracellular matrix (ECM) components and connective tissues. Previous studies have indicated that efficient alveolar epithelial cell repair is critical for normal healing without fibrosis [1,2]. Previously, local tissue myofibroblasts were thought to be the primary source of ECM components following injury [3]. However, ECM components are now thought to derive from multiple sources [4], such as resident lung fibroblasts, bone marrow-derived fibrocytes, and other circulating fibroblast progenitor cells and the epithelial–mesenchymal transition (EMT) [4,5,6,7]. Idiopathic pulmonary fibrosis (IPF) is the most common idiopathic interstitial lung disease but has the worst prognosis, with a median survival time of 3–4 years [8]. The survival rate has not been improved by the recent introduction of two antifibrotic drugs; thus, lung transplantation remains the only effective treatment [9]. Emerging evidence suggests that the EMT process is a major event during IPF pathogenesis [9,10,11].

Growth arrest-specific protein 6 (Gas6) is a secreted protein that contains an N-terminal gamma-carboxyglutamic acid (Gla) domain, followed by four epidermal growth factor (EGF)-like repeats, and a large C-terminal receptor binding domain [12,13]. Gas6 is a common ligand of the Tyro3/Axl/Mer (TAM) receptor subfamily [14,15]. Gas6 is also thought to act as a bridge between apoptotic cells and the phagocytes that ingest them [16,17]. Gas6-TAM binding is associated with a number of cellular processes, including the regulation of cell survival, inflammation, platelet stabilization [17,18,19,20,21], the maintenance of vascular smooth muscle homeostasis, and cancer progression [22,23,24]. The activation of Axl or Mer by Gas6 in macrophages and dendritic cells modulates Toll-like receptor (TLR)-induced cytokine secretion and inhibits inflammatory responses, either through the synthesis of TLR suppressors [19] or through a downstream pathway that involves phosphatidylinositol 3-kinase (PI3K)/AKT and nuclear factor-kappaB (NF-κB) [25,26]. Data from our previous studies demonstrated that macrophages can be re-programmed by Gas6 to promote epithelial proliferation and wound repair via the paracrine functions of hepatocyte growth factor (HGF), which is induced by the Mer/RhoA/AKT/mitogen-activated protein (MAP) kinase pathway in macrophages [27]. Moreover, we have reported that relatively low levels of HGF can inhibit transforming growth factor (TGF)-β1-induced changes in EMT markers, at both the gene and protein levels [28]. However, the upregulation of HGF promotes carcinogenesis and EMT in hepatocellular carcinomas via AKT and cyclooxygenase-2 (COX-2) pathways [29,30]. Recently, we reported that Gas6 inhibits TGF-β1-induced EMT and signaling in lung epithelial cells, via the activation of Axl or Mer and the COX-2-dependent production of prostaglandin E_2_ (PGE_2_) and prostaglandin D_2_ (PGD_2_) [31]. Based on these findings, here we examined the role of the HGF-signaling pathway during the inhibitory effects of Gas6 on the EMT process, as well as during the inhibitory effects of Gas6 on the migration and invasion of LA-4 and primary mouse alveolar type II (AT II) epithelial cells. We demonstrated that Gas6 induces HGF and c-Met production in lung AT II cells via RhoA signaling and that the HGF pathway mediates the Gas6-induced inhibition of both TGF-β1-induced EMT and the migration and invasion of lung epithelial cells.

## 2. Materials and Methods

### 2.1. Reagents

Recombinant mouse Gas6 was purchased from R&D Systems (Minneapolis, MN, USA). HGF was purchased from Cayman Chemical (Ann Arbor, MI, USA). Y-27632 (Sigma-Aldrich Chemical Co., St. Louis, MO, USA) and PHA-665752 (Santa Cruz Biotechnology, Santa Cruz, CA, USA) were used as supplied. The gene-specific relative reverse transcription polymerase chain reaction (RT-PCR) kit was obtained from Invitrogen (Carlsbad, CA, USA), and Moloney Murine Leukemia Virus (M-MLV) reverse transcriptase was purchased from Enzynomics (Hanam, Korea). The enzyme immunoassay (EIA) kits for HGF were obtained from R&D Systems. The antibodies used in this study detected the following proteins: E-cadherin, α-SMA (Abcam, Cambridge, MA, USA), N-cadherin, c-Met (Cell Signaling Technology, Beverly, MA, USA), HGF-α, phospho-extracellular signal-related kinase (ERK)1/2, ERK, phospho-AKT Ser473, AKT (Santa Cruz Biotechnology), and α-tubulin (Sigma-Aldrich).

### 2.2. Cell Lines and Culture

LA-4 cells were purchased from American Type Culture Collection (ATCC). LA-4 cells were grown in F12K medium (Lonza, Basel, Switzerland), containing 15% heat-inactivated fetal bovine serum (FBS), at 37 °C in 5% CO_2_.

### 2.3. Isolation and Culture of Primary Cells

Specific pathogen free, male BALB/c mice (Orient Bio, Sungnam, Korea) weighing 20–22 g was used for isolation of primary AT II cells. The Animal Care Committee of the Ewha Medical Research Institute approved the experimental protocol (ESM17-0376). Mice were cared for and handled in accordance with the National Institute of Health (NIH) Guide for the Care and Use of Laboratory Animals. Primary murine AT II cells were isolated and purified using a modification of previously published methods [32,33]. The purity of AT II cells was typically >90%, as assessed using prosurfactant protein C (pro-SP-C) immunofluorescence staining [34].

### 2.4. Incubation of Epithelial Cells

LA-4 cells were plated in 6-well culture plates (2 × 10^5^ cells/well) and cultured overnight in 200 µL RPMI 1640 or Dulbecco’s Modified Eagle Medium (DMEM), containing 10% FBS. Primary AT II cells were plated and cultured on type 1 collagen-coated culture plates (1 × 10^6^ cells/well) for 48 h. Cells were treated for 20 h with 400 ng/mL Gas6, and then 10 ng/mL TGF-β1 (R&D Systems Inc) was used to treat the cells for 48 or 72 h [28]. In some experiments, 30 μM Y-27632 was used to inhibit Rho kinase. The specific inhibitor was added 1 h before cells were treated with Gas6. In addition, 250 nM PHA-665752 was used to antagonize c-Met. The receptor antagonist was added 1 h before the addition of TGF-β1.

### 2.5. Transient Transfections

LA-4 cells were transiently transfected with 1 µg/mL of siRNA that specifically targeted either RhoA, Axl, or Mer or with control siRNA (Bioneer, Seoul, Korea), using 5 µL of siRNA transfection reagent (Genlantis, San Diego, CA, USA) according to the manufacturer’s protocol. The siRNA sequences used for targeting genes were as follows (gene: sense, antisense). *RhoA* (#1): 5′-GAAGUCAAGCAUUUCUGUCTT A-3′, 5′-GACAGAAAUGCUUGACUUCTT-3′; *RhoA* (#2): 5′-GUCUCAUGUUAGUUACCUUTT-3′, 5′-AAGGUAACUAACAUGAGACTT-3′; *Axl* (#1): 5′-GAGAUGGACAGAUCCUAGA-3′, 5′-UCUAGGAUCUGUCCAUCUC-3′; *Axl* (#2): 5′-CACACACUCAAGAAUCCAATT-3′, 5′-UUGGAUUCUUGAGUGUGUGTT-3′; *Mer* (#1): 5′-CACAGUUUUAUCCUGAUGA-3′, 5′-UCAUCAGGAUAAAACUGUG-3′; *Mer* (#2): 5′-UGACAGAAACCUUCUGGUUTT-3′, 5′-AACCAGAAGGUUUCUGUCATT-3′. Cells were incubated in serum-free medium for 24 h prior to siRNA experiments. None of the siRNAs used had any significant effects on cell viability.

### 2.6. Immunoblot Analysis

To detect the expression of epithelial and mesenchymal markers, cells were lysed in lysis buffer containing 0.5% Triton X-100 and resolved on a 10% sodium dodecyl sulfate polyacrylamide gel electrophoresis (SDS-PAGE) gel prior to transfer onto nitrocellulose membranes. Membranes were blocked at room temperature with Tris-buffered saline containing 3% BSA and then incubated at room temperature with various anti-mouse primary antibodies and probed with an anti-mouse horse radish peroxidase (HRP)-conjugated secondary antibody. Bands were visualized using enhanced chemiluminescence.

### 2.7. Quantitative Real-Time Polymerase Chain Reaction (qPCR)

Gene expression was analyzed by real-time qPCR on a StepOnePlus system (Applied Biosystems, Life Technologies, Carlsbad, CA, USA). For each qPCR assay, a total of 50 ng cDNA was used. Primer sets for the PCR-based amplifications were designed using Primer Express software. The primers used were as follows (name: forward primer, reverse primer). For mice, *HGF*: 5′-CACCCCTTGGGAGTATTGTG-3′, 5′-GGGACATCAGTCTCATTCAC-3; *c-Met*; 5′-GTGCCAAGCTACCAGT-3′, 5′-CTTCGTACAAGGCGTCT-3′; *Cdh1*: 5′-GCACTCTTCTCCTGGTCCTG-3′, 5′-TATGAGGCTGTGGGTTCCTC-3′; *Cdh2*: 5′-CCTCCAGAGTTTACTGCCATGAC-3′, 5′-CCACCACTGATTCTGTATGCCG-3′; *Acta2*: 5′-CCACCGCAAATGCTTCTAAGT-3′, 5′-GGCAGGAATGATTTGGAAAGG-3′; *Snai1*: 5′-CCCAAGGCCGTAGAGCTGA-3′, 5′-GCTTTTGCCACTGTCCTCATC-3′; *Snai2*: 5′-ATCCTCACCTCGGGAGCATA-3′, 5′-TGCCGACGATGTCCATACAG-3′; *Zeb1*: 5′-ATTCAGCTACTGTGAGCCCTGC-3′, 5′-CATTCTGGTCCTCCACAGTGGA-3′; *Zeb2*: 5′-GCAGTGAGCATCGAAGAGTACC-3′, 5′-GGCAAAAGCATCTGGAGTTCCAG-3′; *Twist1*: 5′-TCGACTTCCTGTACCAGGTCCT-3′, 5′-CCATCTTGGAGTCCAGCTCG-3′; and *Hprt*: 5′-CCAGTGTCAATTATATCTTCAAC-3′, 5′-CAGACTGAAGAGCTACTGTAATG-3′. The cDNA abundances were normalized to that for hypoxanthine-guanine phosphoribosyltransferase (*Hprt*) [35] and are presented as the fold-change in abundance compared with the appropriate controls.

### 2.8. RhoA Activity Assay

RhoA activity was measured in LA-4 cell lysates using an enzyme-linked immunosorbent assay (ELISA)-based RhoA activation assay Biochem Kit (G-LISA, Cytoskeleton, Denver, CO, USA) according to the manufacturer’s instructions. In brief, cell lysates were added to the RhoA-GTP affinity plate that was coated with the Rhotekin binding domain of RhoA for 30 min. The active GTP-bound form of RhoA was measured using indirect immunodetection followed by a colorimetric reaction at 490 nm on a microplate spectrophotometer.

### 2.9. Enzyme-Linked Immunosorbent Assay (ELISA) Measurement

Culture supernatants were collected, and HGF levels were measured by a specific ELISA kit according to the manufacturer’s instructions (R&D Systems, Minneapolis, MN, USA).

### 2.10. Migration and Invasion Assays

Cell migration and invasion were tested using Transwell chambers (Corning Inc., Corning, NY, USA) coated with 10 μg/mL fibronectin and 300 μg/mL Matrigel matrix, respectively. Pre-incubated primary mouse AT II cells or LA-4 cells (5 × 10^4^ cells/well for the migration assay and 5 × 10^4^ cells/well for the invasion assay) in the absence or presence of TGF-β1 (10 ng/mL) were plated in replicate wells in serum-free RPMI in the upper chambers and in RPMI 1640 supplemented with 10% FBS placed in the bottom wells at 37 °C for 48 or 72 h. The non-migrated or non-invaded cells on the upper surface of the membrane were removed with a cotton swap. Migrated cells on the lower surface were fixed with 4% paraformaldehyde and stained using 0.1% crystal violet. Three random microscopic fields (10× magnification) were photographed and counted. All experiments were performed in triplicate.

### 2.11. Statistical Analysis

Data are expressed as the mean ± standard error of the mean (SEM). Analysis of variance was used for multiple comparisons, and Tukey’s post hoc test was used where appropriate. The Student’s *t*-test was used to compare two sample means. A *P*-value of less than 0.05 was considered to be statistically significant. All data were analyzed using JMP software (version 3, SAS Institute, Cary, NC, USA).

## 3. Results

### 3.1. Growth Arrest-Specific Protein 6 (Gas6)/Axl or Mer Signaling Induces RhoA-Dependent Hepatocyte Growth Factor (HGF) Secretion and c-Met Expression in Lung Epithelial Cells

**Figure 1:**
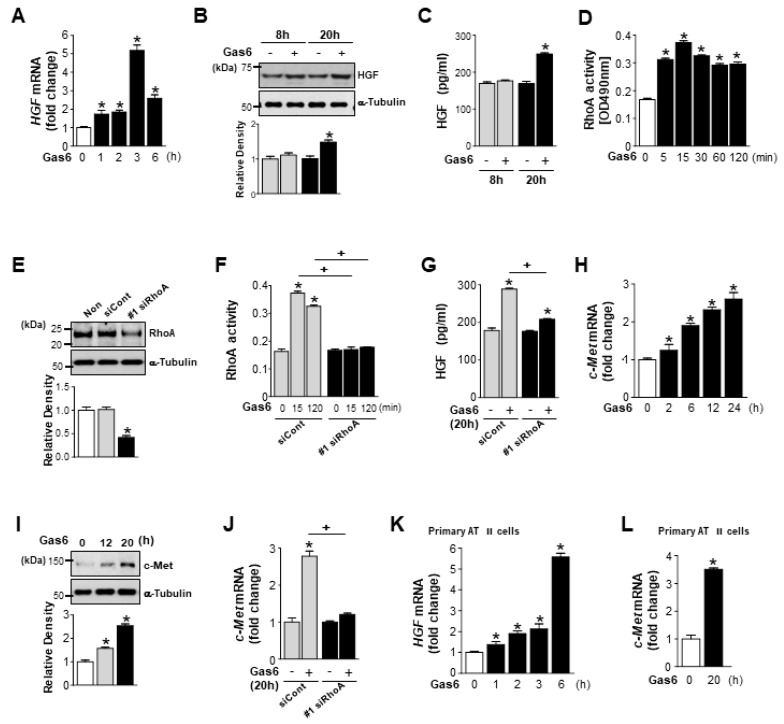
Growth arrest-specific protein 6 (Gas6) induces RhoA-dependent hepatocyte growth factor (HGF) production and c-Met expression in lung epithelial cells. (**A**–**D**) LA-4 cells were stimulated with 400 ng/mL Gas6 for the indicated times. (**A**) quantitative real-time polymerase chain reaction (qPCR) analysis of *HGF* mRNA levels. (**B**) Immunoblot analysis of HGF levels in LA-4 cells. (**C**) Enzyme-linked immunosorbent assay (ELISA) examining HGF levels in conditioned medium (CM) from LA-4 cells. (**D**) RhoA activity in LA-4 cells after Gas6 treatment. (**E**–**G**,**J**) LA-4 cells were transfected with *RhoA* or control small interfering RNA (siRNA) (#1 siRhoA or siCont) for 24 h. (**E**) Immunoblot analysis of RhoA levels in LA-4 cells. (**F**) RhoA activity in LA-4 cells after 400 ng/mL Gas6 treatment for the indicated times. (**G**) ELISA examining HGF levels in CM from LA-4 cells 24 h after 400 ng/mL Gas6 treatment. (**H**) qPCR analysis of *c-Met* mRNA levels after Gas6 treatment for the indicated times. (**I**) Immunoblot analysis of c-Met levels in LA-4 cells after Gas6 treatment for the indicated times. (**J**) qPCR analysis of *c-Met* mRNA levels in LA-4 cells transfected with *RhoA* or control siRNA for 24 h prior to Gas6 treatment for 20 h. (**K**,**L**) Primary AT II epithelial cells were stimulated with 400 ng/mL Gas6 for the indicated times. qPCR analysis of *HGF* mRNA levels in K and *c-Met* mRNA levels in I, in primary AT II cells 20 h after Gas6 treatment. Values represent the mean ± standard error of the mean (SEM) of three independent experiments. * *p* < 0.05 compared with control; ^+^ *p* < 0.05, as indicated.

We first examined whether Gas6 induces RhoA-dependent HGF secretion in LA-4 lung epithelial-like cells. Treatment with Gas6 induced HGF mRNA expression, which peaked after 3 h and declined to half of the peak level after 6 h, in LA-4 cells (Figure 1A). Immunoblot analysis of LA-4 cell lysates using an anti-HGFα-chain antibody indicated that intracellular HGF protein expression was also enhanced by Gas6 treatment (Figure 1B). HGF secretion, as measured by ELISA, significantly increased in LA-4 cells after Gas6 treatment compared with basal levels of secretion (Figure 1C). RhoA activity was significantly enhanced 5 min after Gas6 treatment, was enhanced for a further 15 min after treatment, and then slightly decreased up to 2 h after treatment (Figure 1D). To inhibit RhoA/Rho kinase signaling, LA-4 cells were transfected with two *RhoA*-specific siRNAs for 24 h. *RhoA* knockdown with two siRNAs (approximately 60% reduction of RhoA protein, Figure 1E; Figure S1A) prevented Gas6-induced increases in RhoA activity and HGF production (Figure 1F,G; Figure S1B,C), indicating that HGF secretion is RhoA-dependent. Interestingly, both the mRNA and protein levels of the HGF receptor c-Met were enhanced up to 20–24 h after Gas6 treatment (Figure 1H,I). *RhoA* knockdown resulted in reduced *c-Met* mRNA expression in LA-4 cells (Figure 1J; Figure S1D). Increases in *HGF* and *c-Met* mRNA expression levels were also induced by Gas6 treatment in mouse primary AT II epithelial cells (Figure 1K,L).

### 3.2. Axl and Mer Receptor Tyrosine Kinases Mediate Gas6-Induced RhoA Activity, and Expression of HGF and c-Met in LA-4 Cells

**Figure 2:**
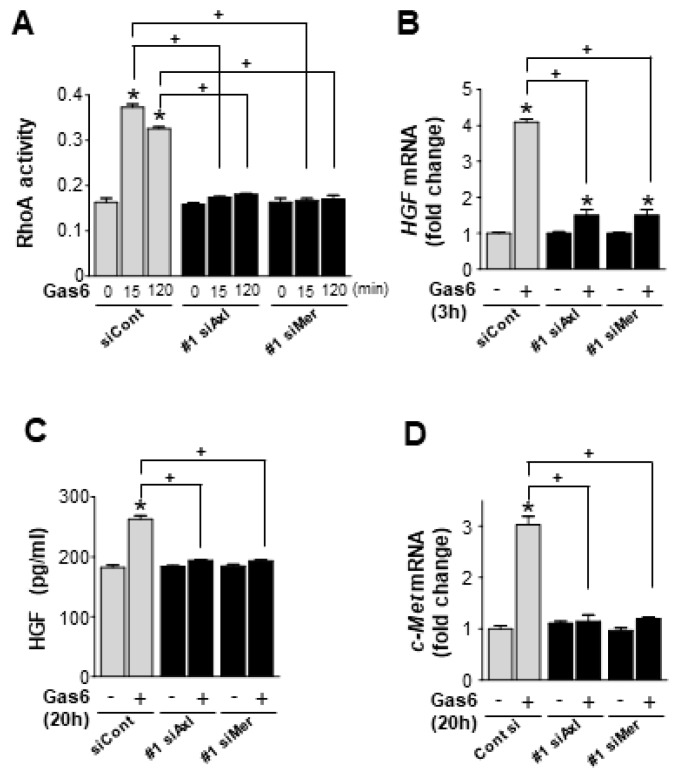
Axl or Mer is involved in the Gas6-mediated enhanced RhoA activity and increased expression levels of HGF and c-Met in lung epithelial cells. (**A**–**D**) LA-4 cells were transfected with siRNAs of *Axl*, *Mer*, or control (#1 siAxl, #1 siMer or siCont) for 48 h and then stimulated with 400 ng/mL Gas6. (**A**) RhoA activity in LA-4 cells after 400 ng/mL Gas6 treatment for the indicated times. (**B**,**D**) qPCR analysis of HGF mRNA levels 3 h after Gas6 treatment and c-Met mRNA levels 20 h after Gas6 treatment, respectively, in LA-4 cells. (**C**) ELISA examining HGF levels in CM from LA-4 cells 24 h after Gas6 treatment. Values represent the mean ± SEM of three independent experiments. * *p* < 0.05 compared with control; ^+^ *p* < 0.05, as indicated.

Next, we examined the contributions made by Axl and Mer signaling to Gas6-induced RhoA activity and HGF expression in LA-4 cells, using two kinds of *Axl* or *Mer*-specific siRNAs. LA-4 cells were transfected with either an *Axl*- or *Mer*-specific siRNA or a negative control siRNA and cultured for 48 h. With Axl or Mer protein knockdown (Figure S2A,B) [31], Gas6-induced RhoA activity in LA-4 cells was substantially suppressed (Figure 2A; Figure S2C). Concomitantly, the Gas6-induced increase in HGF expression levels was prevented by the silencing of either *Axl* or *Mer* RNA, at both the gene and protein levels (Figure 2B,C; Figure S2D,E). Moreover, the specific siRNAs targeting *Axl* and *Mer* prevented the Gas6-induced increase in *c-Met* expression at the gene level (Figure 2D; Figure S2F). Taken together, these data suggest that the Gas6/Axl or Mer signaling pathway induces RhoA-dependent HGF and c-Met expression, at both the gene and protein levels, in lung epithelial cells.

### 3.3. RhoA is Involved in the Gas6-Induced Inhibition of Epithelial–Mesenchymal Transition (EMT) Marker Changes in LA-4 Cells

**Figure 3:**
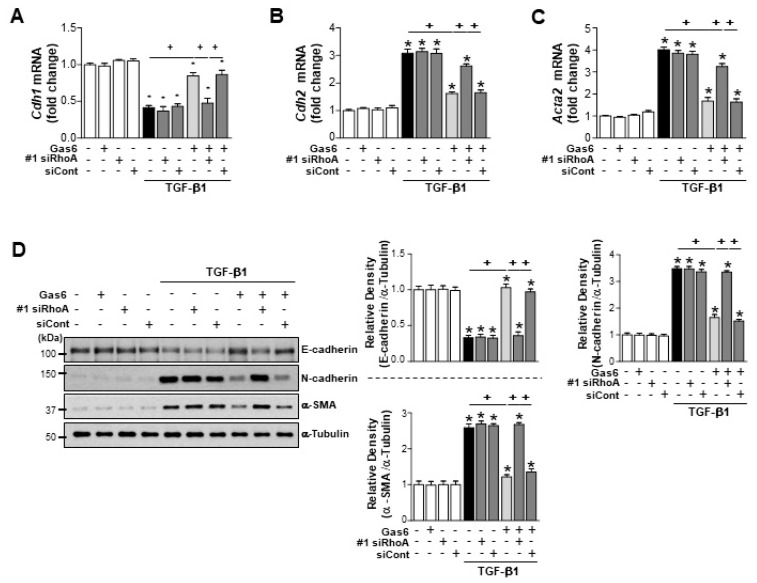
RhoA is involved in the Gas6-induced inhibition of epithelial–mesenchymal transition (EMT) in LA-4 cells. LA-4 cells were transfected with *RhoA*-specific or control siRNAs (#1 siRhoA or siCont) for 24 h prior to treatment with 400 ng/mL Gas6 for 20 h and then stimulated with 10 ng/mL transforming growth factor (TGF)-β1 for 48 h. (**A**–**C**) qPCR analysis of the mRNA levels of EMT markers and EMT-regulating transcription factors. (**D**) Immunoblots of total cell lysates were performed using anti-E-cadherin, anti-N-cadherin, or anti-α-SMA antibodies. Densitometry of the relative abundances of the indicated EMT markers. Alpha-tubulin was used as a control. Values represent the mean ± SEM of three independent experiments. * *p* < 0.05 compared with control; ^+^ *p* < 0.05, as indicated. Results are representative of three independent experiments.

We examined whether RhoA-dependent HGF signaling is involved in the anti-EMT effects observed following Gas6 treatment. *RhoA* siRNA was transfected in LA-4 cells for 48 h, then cells were treated with Gas6 for 20 h before being treated with TGF-β1 for 48 h. Transfection with two *RhoA* siRNAs prevented the Gas6-induced inhibition of TGF-β1-induced EMT, including reducing the levels of E-cadherin loss and reducing the synthesis of N-cadherin and α-SMA at both the gene and protein levels (Figure 3A–D; Figure S3A–D). These data suggest that RhoA signaling mediates Gas6-induced EMT inhibition in LA-4 cells.

### 3.4. RhoA is Involved in the Inhibition of Phospho-Extracellular Signal-Related Kinase (ERK1/2) and AKT Activation and Reduction of EMT-Regulating Transcription Factor Expression by Gas6 in LA-4 Cells

**Figure 4:**
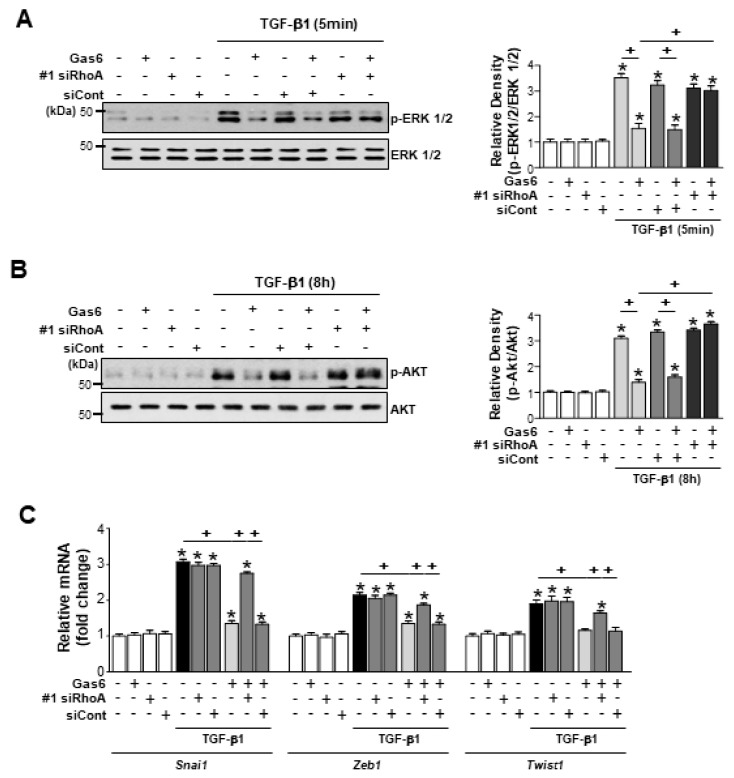
RhoA is involved in the Gas6-induced inhibition of non-Smad signaling pathways and EMT transcription factors in LA-4 cells. LA-4 cells were transfected with *RhoA*-specific or control siRNAs (#1 siRhoA or siCont) for 24 h prior to treatment with 400 ng/mL Gas6 for 20 h and then stimulated with 10 ng/mL TGF-β1 for the indicated times or 48 h. (**A**,**B**) Representative immunoblots of LA-4 cell lysates were performed with anti-total/phosphorylated extracellular signal-related kinase (ERK)1/2 antibodies in **A** and with anti-total/phosphorylated AKT (Ser473) antibodies in (**B**). (**C**) qPCR analysis of the mRNA levels of EMT-regulating transcription factors. Values represent the mean ± SEM of three independent experiments. * *p* < 0.05; compared with control; ^+^ *p* < 0.05, as indicated. Results are representative of three independent experiments.

*RhoA* siRNA completely prevented the TGF-β1-induced activation of non-Smad signaling pathways, such as the phosphorylation of ERK1/2 and AKT, in LA-4 cells treated with Gas6, whereas negative control siRNA had no effect (Figure 4A,B; Figure S4A,B). *RhoA* siRNA also prevented the downregulation of *Snai1*, *Zeb1*, and *Twist1* expression levels in LA-4 cells following Gas6 treatment (Figure 4C; Figure S4C). These data suggest that RhoA signaling is required for Gas6-induced inhibition of the phosphorylation of ERK1/2 and AKT and reduction of EMT-regulating transcription factor expression in LA-4 cells.

### 3.5. The Rho Kinase is Involved in the Inhibition of EMT Marker Changes and Reduction of EMT-Regulating Transcription Factor Expression by Gas6 in Lung Epithelial Cells

**Figure 5:**
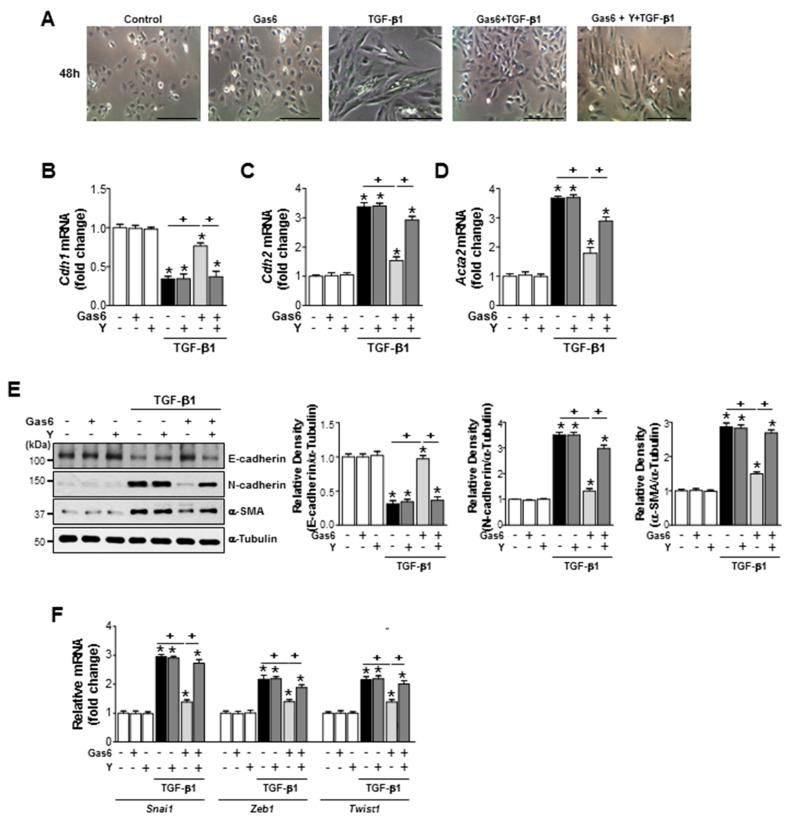
Rho kinase is involved in the Gas6-induced inhibition of EMT in LA-4 cells. LA-4 cells were treated with 30 µM Y-27632 for 1 h prior to treatment with 400 ng/mL Gas6 for 20 h and then stimulated with 10 ng/mL TGF-β1 for 48 h. (**A**) Morphological changes in the cells were examined by phase-contrast microscopy (Scale bars = 50 µm). (**B**–**D** and **F**) qPCR analysis of the mRNA levels of EMT markers and EMT-regulating transcription factors. (**E**) Immunoblots of total cell lysates were performed using anti-E-cadherin, anti-N-cadherin, or anti-α-SMA antibodies. Densitometry of the relative abundances of the indicated EMT markers. Alpha-tubulin was used as a control. Values represent the mean ± SEM of three independent experiments. * *p* < 0.05 compared with control; ^+^ *p* < 0.05, as indicated. Results are representative of three independent experiments.

**Figure 6:**
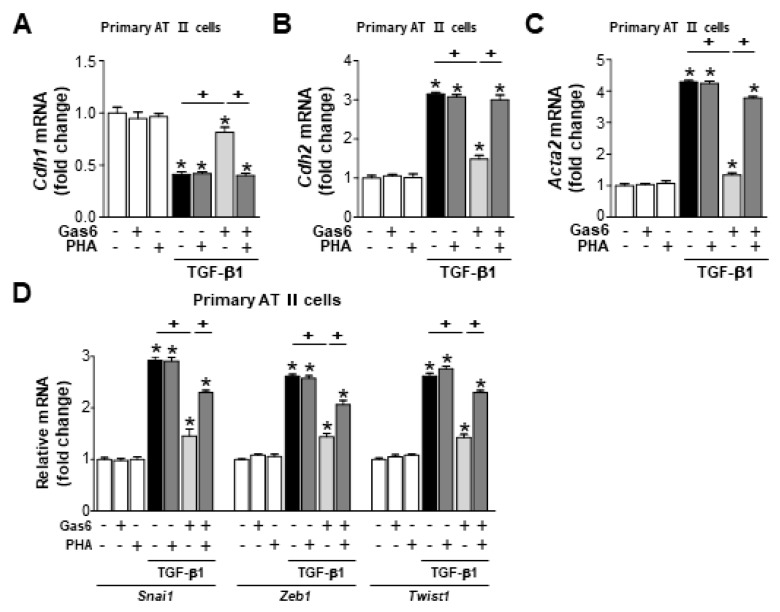
Rho kinase is involved in the Gas6-induced inhibition of EMT markers and EMT transcription factors in primary alveolar type II cells. Primary alveolar type II (AT II) cells were treated with 30 µM Y-27632 for 1 h prior to treatment with 400 ng/mL Gas6 for 20 h and then stimulated with 10 ng/mL TGF-β1 for 48 h. (**A**–**D**) qPCR analysis of the mRNA levels of EMT markers and EMT transcription factors. Values represent the mean ± SEM of three independent experiments. * *p* < 0.05 compared with control; ^+^ *p* < 0.05, as indicated.

Treatment with 30 µM Y-27632, a pharmacologic inhibitor of Rho kinase, 1 h before Gas6 treatment also prevented the anti-EMT effects of Gas6, including TGFβ1-induced spindle-like cellular morphologic changes (Figure 5A), EMT marker expression at both the gene and protein levels (Figure 5B–E), and the mRNA expression of EMT-regulating transcription factors, such as *Snai1*, *Zeb1*, and *Twist1*, in LA-4 cells (Figure 5F). The Gas6-induced reductions in TGF-β1-induced EMT marker (Figure 6A–C) and EMT-regulating transcription factor (Figure 6D) gene expression levels were also prevented in primary AT II epithelial cells treated with Y-27632. Taken together, these data suggest that the RhoA/Rho kinase signaling is involved in anti-EMT effects of Gas6 in LA-4 cells and primary AT II epithelial cells.

### 3.6. c-Met Signaling is Involved in the Gas6-Induced Inhibition of EMT in LA-4 Cells and Primary AT II Cells

**Figure 7:**
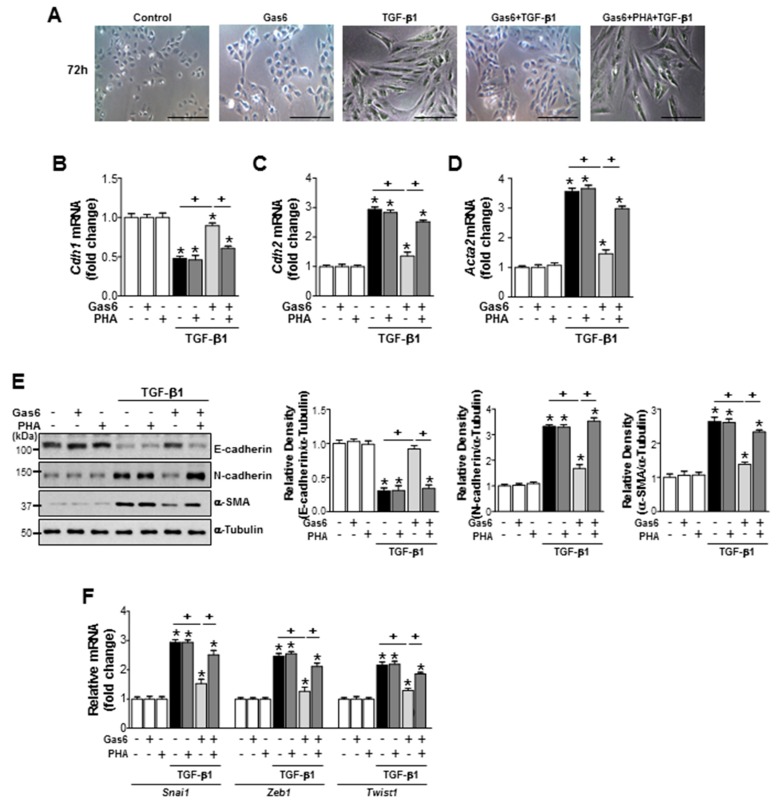
c-Met signaling mediates the Gas6-induced inhibition of EMT in LA-4 cells. LA-4 cells were treated with 400 ng/mL Gas6 for 20 h and then stimulated with 10 ng/mL TGF-β1 for 72 h, with or without 250 nM PHA-665752. (**A**) Morphological changes in the cells were examined by phase-contrast microscopy (Scale bars = 50 µm). (**B**–**D**,**F**) qPCR analysis of the mRNA levels of EMT markers and EMT-inducing transcription factors. (**E**) Immunoblots of total cell lysates were performed using anti-E-cadherin, anti-N-cadherin, or anti-α-SMA antibodies. Densitometry of the relative abundances of the indicated EMT markers. Alpha-tubulin was used as a control. Values represent the mean ± SEM of three independent experiments. * *p* < 0.05 compared with control; ^+^ *p* < 0.05, as indicated. Results are representative of three independent experiments.

**Figure 8:**
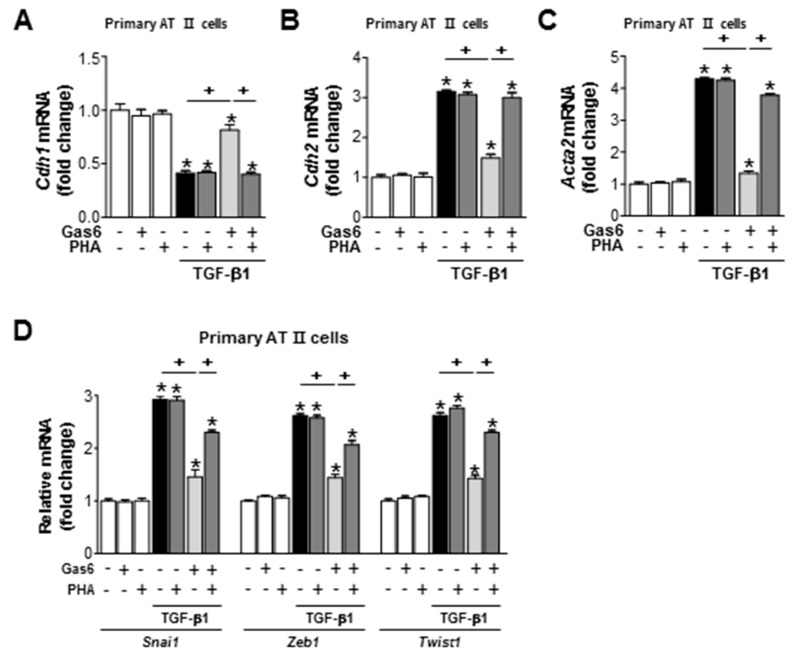
c-Met signaling mediates the Gas6-induced inhibition of EMT markers and EMT transcription factors in primary alveolar type II cells. Primary AT II cells were treated with 400 ng/mL Gas6 for 20 h and then stimulated with 10 ng/mL TGF-β1 for 72 h, with or without 250 nM PHA-665752. (**A**–**D**) qPCR analysis of the mRNA levels of EMT markers and EMT-inducing transcription factors. Values represent the mean ± SEM of three independent experiments * *p* < 0.05 compared with control; ^+^ *p* < 0.05, as indicated.

c-Met signaling was inhibited by treating LA-4 cells with the c-Met antagonist PHA-665752 (250 nM) 1 h before TGF-β1 treatment, which also prevented the anti-EMT effects of Gas6, including reversing the spindle-like cellular morphology (Figure 7A), reducing the levels of E-cadherin loss, reducingα-SMA and N-cadherin synthesis at both the gene and protein levels (Figure 7B–E), and reducing *Snai1*, *Zeb1,* and *Twist1* mRNA expression levels (Figure 7F). In addition, the preventative effects of PHA-665752 on the Gas6-induced inhibition of TGF-β1-induced EMT marker (Figure 8A–C) and EMT-regulating transcription factor (Figure 8D) gene expression levels were also observed in primary AT II epithelial cells. These data suggest that RhoA/Rho kinase-dependent HGF production mediates the anti-EMT effects of Gas6 via c-Met signaling in LA-4 cells and primary AT II epithelial cells.

### 3.7. Treatment of Lung Epithelial Cells with Gas6 Inhibits the Migration and Invasion of Epithelial Cells via RhoA-Dependent HGF Secretion

**Figure 9:**
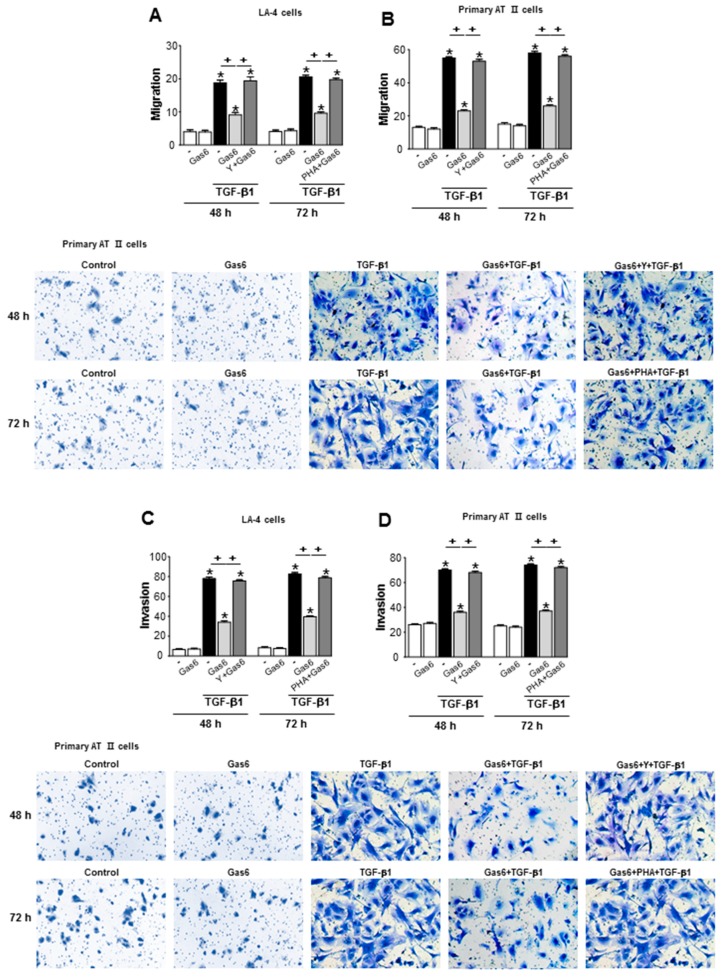
Gas6/RhoA/HGF signaling inhibits the migration and invasion of lung epithelial cells. (**A**–**D**) LA-4 or primary AT II cells were treated with 30 µM Y-27632 for 1 h prior to treatment with 400 ng/mL Gas6 for 20 h and then stimulated with 10 ng/mL TGF-β1 for 48 h. Epithelial cells were treated with 400 ng/mL Gas6 for 20 h and then stimulated with 10 ng/mL TGF-β1 for 72 h, with or without 250 nM PHA-665752. The quantification of migrating or invading cells was performed in Transwell chambers. Primary AT II cells were visualized by phase-contrast microscopy for the analysis of migration (**B** lower) and invasion (**D** lower) abilities, using fibronectin-coated Transwell and Matrigel-coated Transwell plates, respectively. Quantification of migrating cells in A and B upper or invading cells in **C** and **D** upper. Values represent the mean ± SEM of three independent experiments. * *p* < 0.05 compared with control; ^+^ *p* < 0.05, as indicated.

TGF-β1-induced EMT is an important step during epithelial cell migration and invasion toward the interstitial area and the alveolar space during the progression of lung fibrosis [36]. Thus, we examined the role played by RhoA-dependent HGF during the Gas6-induced inhibition of migration and invasion in LA-4 and primary murine AT II cells, using Transwell and Matrigel assays, respectively. Pretreatment with the Rho kinase inhibitor Y27632 (30 µM) or the c-Met antagonist PHA-665752 (250 nM) prevented the Gas6-induced suppression of TGF-β1-induced migration and invasion in LA-4 cells (Figure 9A,C) and primary AT II cells (Figure 9B,D). These data indicated that RhoA/Rho kinase-dependent HGF/c-Met signaling prevents the EMT process and migration and invasion in LA-4 and primary murine AT II cells.

## 4. Discussion

RhoA/PI3K/AKT/MAP kinase signaling following Gas6/Mer engagement promotes epithelial cell growth and wound repair via the upregulation of HGF in macrophages [27]. In the present study, we demonstrated that Gas6 treatment enhances RhoA activity, resulting in enhanced HGF mRNA and protein levels, in LA-4 cells. This result was confirmed using *RhoA*-specific siRNA, which demonstrated that HGF secretion following Gas6 stimulation was RhoA-dependent in LA-4 cells. Moreover, Gas6 treatment increased c-Met expression at the mRNA and protein levels in LA-4 cells, in a time-dependent manner. RhoA-dependent increases in the mRNA expression levels of *HGF* and *c-Met* were also confirmed in primary AT II cells. Both *Axl*- and *Mer*-specific siRNA prevented the Gas6-induced increases in RhoA activity, *HGF* mRNA expression levels, HGF secretion, and *c-Met* mRNA expression levels in LA-4 cells, indicating that Gas6-Axl or -Mer signaling activates a RhoA-dependent pathway, which consequently results in the induction of *HGF* and *c-Met* expression.

Exogenous treatment with 400 ng/mL HGF inhibited TGF-β1-induced EMT processes in LA-4 cells [28]. Previously, we also reported that the pretreatment of LA-4 cells with Gas6 blocked the non-Smad TGF-β1 signaling pathways, such as ERK1/2 and AKT pathways, but not the Smad and p38 MAP kinase pathways, and, consequently, downregulated the mRNA expression levels of EMT-regulating transcription factors, such as *Snai1/2*, *Zeb1/2*, and *Twist1*, in LA-4 cells [31]. In addition, we investigated whether the effects of Gas6 could be prevented by inhibiting RhoA/Rho kinase activity, using either *RhoA* siRNA or the Rho kinase inhibitor Y27632. The siRNA-mediated knockdown of *RhoA* prevented the inhibitory effects of Gas6 on TGF-β1-induced EMT, including reducing the levels of E-cadherin loss and N-cadherin and α-SMA synthesis at the gene and protein levels. *RhoA* knockdown also prevented the Gas6-induced inhibition of non-Smad TGF-β1 signaling pathways, such as ERK1/2 and AKT, and EMT-regulating transcription factors, including *Snai1*, *Zeb1,* and *Twist1*, in LA-4 cells. Similarly, the Gas6-induced inhibition of the EMT process could be prevented by treatment with the RhoA kinase inhibitor Y27632 in both LA-4 cells and primary AT II epithelial cells. Therefore, these data suggest that RhoA/Rho kinase signaling is involved in the inhibitory effects induced by Gas6 treatment against non-Smad TGF-β1 signaling and EMT processes in lung alveolar epithelial cells. Moreover, the c-Met antagonist (PHA-665752) strongly inhibited the Gas6-induced inhibition of TGF-β1-induced EMT processes in both LA-4 cells and primary AT II cells. These data suggested that c-Met activation may play an important role in limiting TGF-β1-induced EMT in LA-4 cells pretreated with Gas6. However, these results do not preclude the involvement of other molecular mechanisms that might affect EMT, including the upregulation of the Smad co-repressor SnoN, the upregulation of Smad7, an inhibitor of the TGF-β1 signaling pathway, or the inhibition of integrin-linked kinase induction [37,38,39].

However, many studies have revealed that very high concentrations of HGF (i.e., greater than 10 ng/mL) can induce EMT and increases the tumor invasiveness of certain types of cancer cells, such as gastric cancer cells, breast cancer cells, and LNCaP prostate cancer cells [29,30,40,41,42]. Although c-Met is important for the control of tissue homeostasis under normal physiological conditions, it has also been found to be aberrantly activated in human cancers via gene mutations and amplifications or protein overexpression [43]. In addition, c-Met controls multiple biological functions, including proliferation, survival, motility and invasion which, when dysregulated by aberrant c-Met activation, can lead to both tumor growth and the metastatic progression of cancer cells.

EMT is characterized by the enhanced migratory and invasive potential of cells. Fibroblasts and myofibroblasts from IPF patients have been shown to have distinct properties [44], including the ability to invade the ECM in the manner of metastatic cancer cells [45]. Consistent with our previous findings [31], treatment with Gas6 inhibited the TGF-β1-induced migration and invasion of both LA-4 cells and primary AT II cells. We further demonstrated that the inhibition of RhoA or c-Met signaling, using Y27632 or by PHA-665752, respectively, prevented the observed anti-migration and invasive effects following Gas6 treatment.

Interestingly, the positive cross-talk between COX-2/PGE_2_ and HGF/c-Met signaling pathways was proposed in macrophages in response to apoptotic cells [46]. It is likely that the findings from our previous [31] and present studies, taken together, suggest that RhoA-dependent HGF and the COX-2-derived PGE_2_ and PGD_2_ signaling pathways may be linked positively, contributing to the anti-EMT and anti-fibrotic effects of Gas6.

## 5. Conclusions

We propose a new model for epithelial cell homeostasis, in which the Gas6/Axl or Mer signaling pathway-mediated anti-EMT programming results in the prevention of the migration and invasion of alveolar type II cells, which may control the progressive fibrotic reaction via the production of RhoA/Rho kinase-dependent HGF and its specific receptor, c-Met.

## Supplementary Materials

The following are available online at https://www.mdpi.com/2218-273X/9/10/565/s1, Figure S1: Gas6 induces RhoA activity, HGF production and c-Met expression in lung epithelial cells, Figure S2: Gas6/Axl or Mer signaling enhances RhoA activity and expression levels of HGF and c-Met in lung epithelial cells, Figure S3: Gas6 induces inhibition of EMT in LA-4 cells via RhoA, Figure S4: Gas6 induces inhibition of non-Smad signaling pathways and EMT transcription factors in LA-4 cells via RhoA.
