# Supplementary material for: RhoA-Dependent HGF and c-Met Mediate Gas6-Induced Inhibition of Epithelial–Mesenchymal Transition, Migration, and Invasion of Lung Alveolar Epithelial Cells
Source: Biomolecules. 2019 Oct 4;9(10):565. doi: 10.3390/biom9100565 (PMC6843420; doi:10.3390/biom9100565)
Supplement: Supplementary file 1 [file biomolecules-09-00565-s001.pdf]

RhoA-dependent HGF and c-Met mediate Gas6-induced inhibition of epithelial-mesenchymal transition, migration, and invasion of lung alveolar epithelial cells

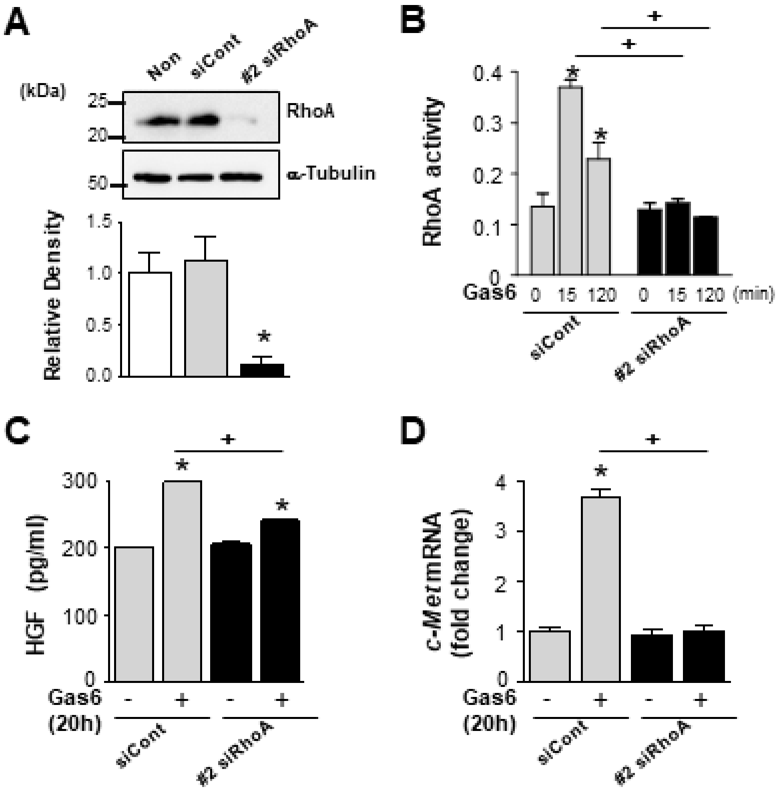

Figure S1. Gas6 induces RhoA activity, HGF production and c-Met expression in lung epithelial cells. (**AD**) LA-4 cells were transfected with *RhoA* or control siRNA (#2 siRhoA or siCont) for 24 h. (**A**) Immunoblot analysis of RhoA levels in LA-4 cells. (**B**) RhoA activity in LA-4 cells after 400 ng/ml Gas6 treatment for the indicated times. (**C**) ELISA examining HGF levels in CM from LA-4 cells 24 h after 400 ng/ml Gas6 treatment. (**D**) qPCR analysis of *c-Met* mRNA levels in LA-4 cells transfected with *RhoA* (#2) or control siRNA for 24 h prior to Gas6 treatment for 20 h. Values represent the mean ± SEM of three independent experiments. *$P < 0.05$ compared with control; $^{+}P < 0.05$, as indicated.

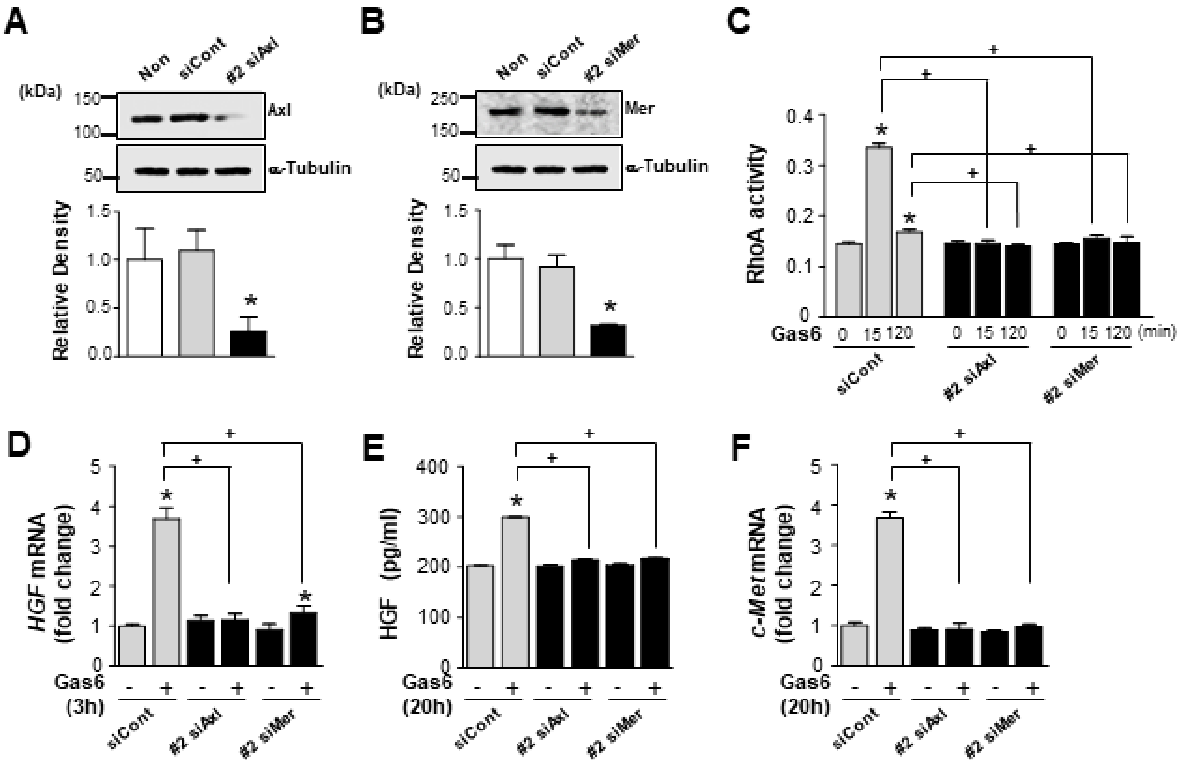

Figure S2. Gas6/Axl or Mer signaling enhances RhoA activity and expression levels of HGF and c-Met in lung epithelial cells. LA-4 cells were transfected with siRNAs of *Axl*, *Mer*, or control (#2 siAxl, #2 siMer, or siCont) for 48 h (**A,B**) and then stimulated with 400 ng/ml Gas6 (**B-F**). (**A,B**) Immunoblot analysis of Axl and Mer levels in LA-4 cells. (**C**) RhoA activity in LA-4 cells after 400 ng/ml Gas6 treatment for the indicated times. (**D,E**) qPCR analysis of HGF mRNA levels 3 h after Gas6 treatment and c-Met mRNA levels 20 h after Gas6 treatment, respectively, in LA-4 cells. (**F**) ELISA examining HGF levels in CM from LA-4 cells 24 h after Gas6 treatment. Values represent the mean ± SEM of three independent experiments. * $p < 0.05$ compared with control; $^{+}p < 0.05$, as indicated.

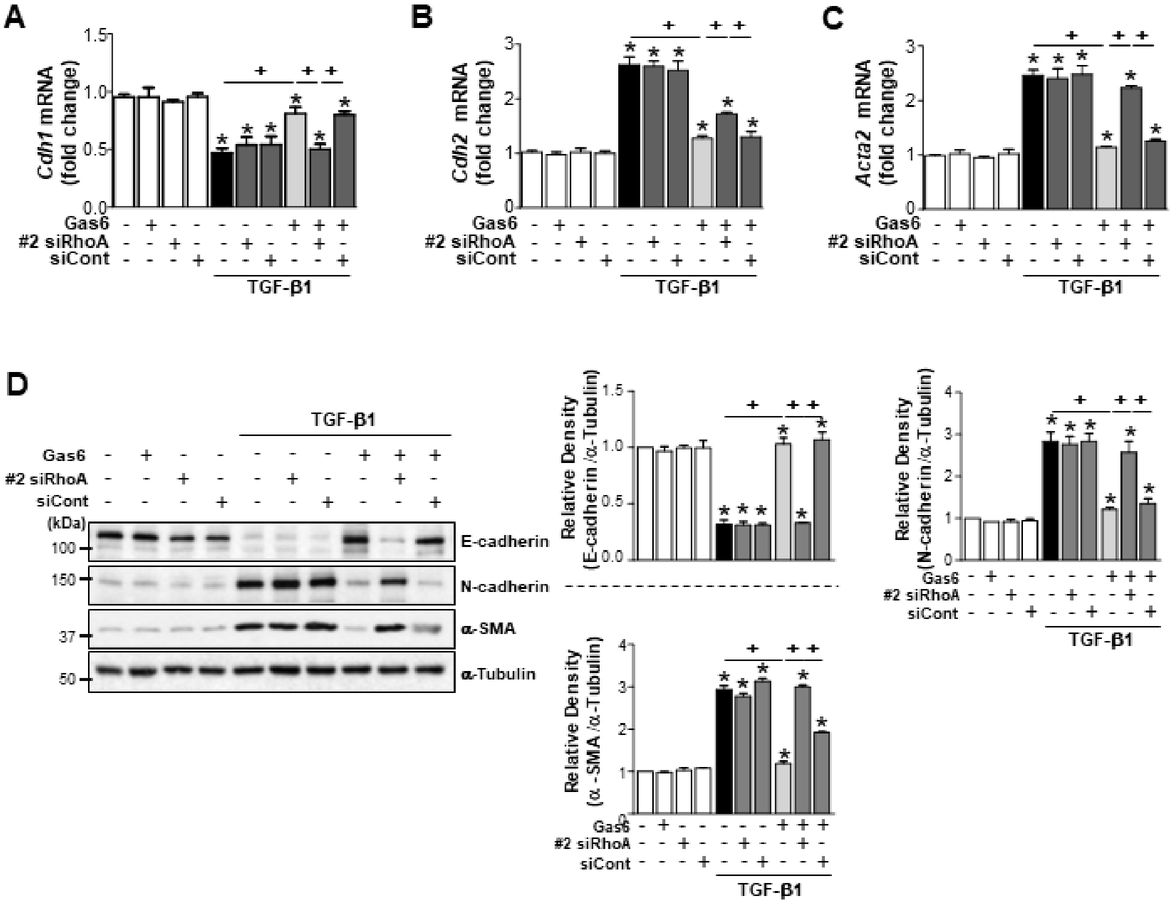

Figure S3. Gas6 induces inhibition of EMT in LA-4 cells via RhoA. LA-4 cells were transfected with *RhoA*-specific or control siRNAs (#2 siRhoA or siCont) for 24 h prior to treatment with 400 ng/ml Gas6 for 20 h and then stimulated with 10 ng/ml TGF-β1 for 48 h. (**A-C**) qPCR analysis of the mRNA levels of EMT markers and EMT-regulating transcription factors. (**D**) Immunoblots of total cell lysates were performed using anti-E-cadherin, anti-N-cadherin, or anti-α-SMA antibodies. Densitometry of the relative abundances of the indicated EMT markers. Alpha-tubulin was used as a control. Values represent the mean ± SEM of three independent experiments. * $p < 0.05$ compared with control; + $p < 0.05$, as indicated. Results are representative of three independent experiments.

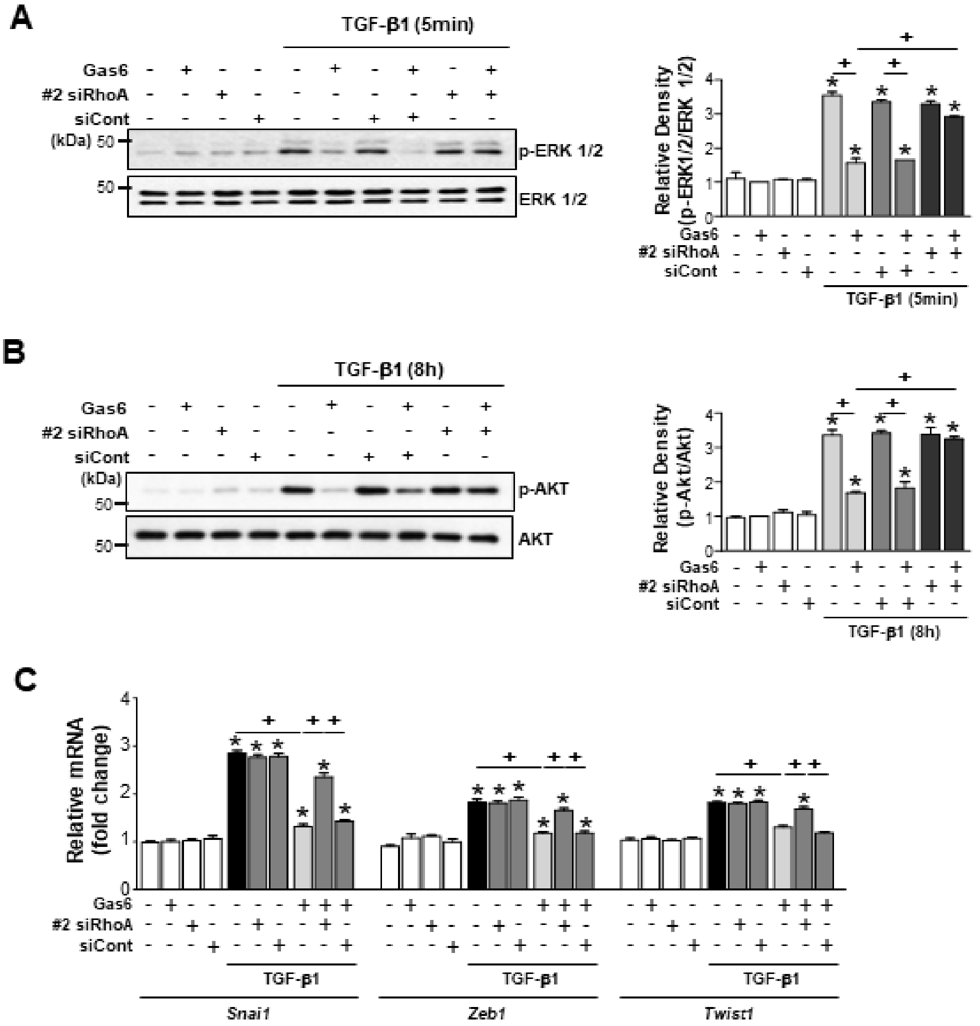

Figure S4. Gas6 induces inhibition of non-Smad signaling pathways and EMT transcription factors in LA-4 cells via RhoA. LA-4 cells were transfected with *RhoA*-specific or control siRNAs (#2 siRhoA or siCont) for 24 h prior to treatment with 400 ng/ml Gas6 for 20 h and then stimulated with 10 ng/ml TGF-β1 for the indicated times or 48 h. (**A**,**B**) Representative immunoblots of LA-4 cell lysates were performed with anti-total/phosphorylated ERK1/2 antibodies in **A** and with anti-total/phosphorylated AKT (Ser473) antibodies in **B**. (**C**) qPCR analysis of the mRNA levels of EMT-regulating transcription factors. Values represent the mean ± SEM of three independent experiments. * $p < 0.05$; compared with control; $^{+}p < 0.05$, as indicated. Results are representative of three independent experiments.
